# Comparative Study on the Influence of Some Medicinal Plants on Diabetes Induced by Streptozotocin in Male Rats

**DOI:** 10.1155/2019/3596287

**Published:** 2019-02-27

**Authors:** Daklallah A. Almalki, Sameera A. Alghamdi, Atef M. Al-Attar

**Affiliations:** ^1^Department of Biology, Faculty of Science and Arts (Qelwah), Albaha University, Saudi Arabia; ^2^Department of Biological Sciences, Faculty of Sciences, King Abdulaziz University, Jeddah, Saudi Arabia

## Abstract

Medicinal plants have played an important role in the treatment of many diseases. Medicinal plants are believed to be well appropriate with the human body and to produce less side influences than the pharmaceuticals. Kingdom of Saudi Arabia has abundant and wide variety of medicinal plants whose therapeutic effects have not been adequately studied. The aim of this study was to investigate the hypoglycemic activities of the extracts of three plant species collected from Albaha region of Saudi Arabia including* Olea oleaster* (Oleaceae family) leaves (OLE),* Juniperus procera* (Cupressaceae family) leaves (JLE), and* Opuntia ficus*-*indica* (Cactaceae family) stems (OSE) on streptozotocin (STZ) diabetic male rats. The animals were distributed into eight groups. The first group was used as normal control. The second group was diabetic control. Diabetic rats of the third, fourth, and fifth groups were supplemented with OLE, JLE, and OSE, respectively. Normal rats of the sixth, seventh, and eighth groups were treated with OLE, JLE, and OSE, respectively. As expected, the mean of body weight was significantly decreased in rats of the second group. Significant increase in the value of serum glucose and decline of insulin value were observed in rats of the second group. Several alterations of lipid and protein profile and oxidative stress markers were noted in diabetic control rats. Severe histopathological alterations of pancreatic tissues were observed in untreated diabetic rats. The obtained results showed that OLE, JLE, and OSE attenuated the physiological and histopathological alterations. These new data indicate that the attenuation influences of OLE, JLE, and OSE attributed to their antioxidant properties confirmed by oxidative stress markers evaluation.

## 1. Introduction

Globally, diabetes mellitus (DM) is one of the most prevalent diseases. DM is a prolonged disease caused by inherited and or acquired deficiency of pancreatic insulin production, or due to the inefficacy of the insulin [1]. DM is characterized by multiple defects in its pathophysiology and abnormalities in carbohydrates, lipids proteins, and metabolism [2–4]. It is evident that this disease leads to hyperglycemia and to many other complications such as hyperlipidemia, hypertension, atherosclerosis, retinopathy, neuropathy, and nephropathy [5–9]. The raising rate of DM depends on several factors such as alterations of people lifestyle and behavior and environment [10]. Globally, the World Health Organization (WHO) reports that the prevalence of DM will be increased and by the year 2025 more 300 million individuals will have DM [11]. The Kingdom of Saudi Arabia has one of the highest percentages of DM in the world.

Therapeutically, medicinal plants have many properties such as the effectiveness, safety, and low cost for many diseases. Remedies from natural products may be effective and safe alternative treatment for DM and its comorbidities. The potential impact of such strategies must be first examined in suitable animal models. Several drugs are used to control DM, however, perfect glucose control is rarely achieved [12]. Recently, encouragement for using medicinal plants as alternative remedies attributed to the elevation of medication cost, synthetic medicine side influences, and lack of full recovery of diabetic patients treated with chemical hypoglycemic agents [13]. Recently, traditional therapies originated from medicinal showed a vital role in the control of DM [14].

The wild olive trees* Olea oleaster*, family Oleaceae, and those olive trees originated in the southwest of Saudi Arabia and eastern Mediterranean. Many experimental investigations showed that olive fruits, leaves, and their compounds possessed a large area of pharmacological and therapeutic properties [15–19]. Juniperus is a plant belonging to Cupressaceae family. Species of Juniperus were traditionally utilized as therapeutic agents for many diseases such as liver and pulmonary sicknesses, wounds treatments, worms of intestine, and ulcers [20, 21].* Juniperus procera *is located in the mountains of the southwest of the Arabian Peninsula and eastern Africa Additionally, Al-Attar et al. [15] showed that* J. procera* leaves extracts possess hepatoprotective properties against hepatic cirrhosis induced by thioacetamide in mice. Cactus (*Opuntia ficus-indica* L), family Cactaceae, is mainly used for fruit production [22]. Several parts of this plant are utilized in the therapy for ulcers, rheumatic pain, wounds, and fatigue [23]. Padilla-Camberos et al. [24] investigated the hypocholesterolemic activity of an aqueous extract of* O. ficus-indica* cladodes (stems) in triton-induced mice. They demonstrated that the extract* O. ficus-indica* showed hypocholesterolemic effect throughout inhibition of pancreatic lipase, and this effect attributed to polyphenolic compounds. Moreover, Smida et al. [25] demonstrated that the administration of this plant extract alleviated the immunotoxicity induced by chlorpyrifos in rats. However, the present investigation was undertaken to assess the influence of* O. oleaster* and* J. procera* leaves and* O. ficus-indica* stems extracts on streptozotocin- (STZ-) induced DM in male rats.

## 2. Material and Methods

### 2.1. Plant Material and Extraction Process

For collecting plant material, the outskirts of Albaha region of Saudi Arabia were chosen. The fresh leaves of* O. oleaster* and* J. procera* and stems of* O. ficus-indica* were collected, washed, and air dried. All dried leaves and stems were powdered and stored at -20°C. The leaves and stems were extracted according to the method of Al-Attar and Abu Zeid [26] with some modifications. 200 g from every plant sample was mixed with 8 liters of hot water for 4 h and slowly boiled for 90 min. All plant solutions were subjected to cooling conditions and every solution was mixed using a suitable electric mixer for 20 min. Subsequently, all solutions were filtered. For obtaining of the dried residues of solutions, an oven at 40°C was used to evaporate the filtrates. Additionally, extraction process was done every two weeks and kept in a fridge for subsequent experimentations.

### 2.2. Animals Model

Male albino rats (*Rattus norvegicus*) weighing 222-256 g were included in this study. The rats were housed in standard cages at 12 h light/12 h dark cycle, temperature of 20±1°C, and humidity (65%). Rats were fed with standard food pellets and water. The experimental animals were left for one week before the start of experiments for acclimatization [8].

### 2.3. Experimental Induction of DM

For DM induction, STZ was used at a single dose of 70 mg/kg body weight. Intraperitoneal injections were used for overnight fasting rats and all injected rats were allowed access to water and food. Additionally, rats were allowed to stable for 4 days and fasting blood glucose concentrations were estimated. Rats with glycemia above 17 mmol/L were included in the study as diabetic model [8].

### 2.4. Treatments

The treatments were initiated on the fifth day after STZ exposure and this is the beginning of the first day of treatments. A dose of 400 mg/kg body weight/day was chosen for all extracts supplementation. The treatments were continued for 5 weeks. The rats were divided into 8 groups comprising 10 animals in each group. Group 1 was utilized as normal control and received saline solution (0.9% NaCl) using intraperitoneal injection. Group 2 was served as untreated diabetic control. Diabetic rats of group 3, 4, and 5 were treated orally with the extracts of* O. oleaster* (OLE),* J. procera* (JLE) and* O*.* ficus-indica* (OSE). Normal (nondiabetic) rats of groups 6, 7, and 8 were received saline solution as group 1 and supplemented orally with OLE, OSE, and JLE, respectively, as groups 3, 4, and 5 [8].

### 2.5. Body Weight Measurement

For body weight evaluation, all experimental animals were weighted at the initiation of the experimental duration and after five weeks. The body weights were recorded at recording time in the morning mentioned by Al-Attar and Zari [27]. Furthermore, for any signs of abnormalities throughout the duration of investigation, the rats were continuously observed

### 2.6. Blood Serum Analysis

After five weeks, rats were fasted for 8 h. Rats were anesthetized using diethyl ether and samples of blood were obtained from orbital venous plexus. Blood serum was separated using cooled centrifugation at 2000 rpm for 10 min. and the serum samples were kept at -80°C. Dimension Vista® 1500 System (USA) was used to measure the levels of selected biochemical parameters including glucose, protein profile (total protein, albumin, and globulin), lipid profile (triglycerides, cholesterol, high density lipoprotein cholesterol (HDL-C), and low density lipoprotein cholesterol, LDL-C), and the enzymatic activities of creatine kinase (CK) and lactate dehydrogenase (LDH). The level serum insulin was estimated according to Judzewitsch et al. [28] method. To evaluate the level of serum very low density lipoprotein cholesterol (VLDL-C), the following equation was used:

(1) $$\begin{array}{c} VLDL\text{-}C=\frac{Triglycerides}{2.175} \end{array}$$

Finally, oxidative stress markers including glutathione (GSH), superoxide dismutase (SOD), malondialdehyde (MDA), and catalase (CAT) were estimated according to the methods of Beutler et al. [29], Nishikimi et al. [30], Ohkawa et al. [31], and Aebi [32], respectively.

### 2.7. Histopathological Examination

After blood collection, all rats were dissected; pancreatic tissues were isolated and fixed in 10% formalin. Fixed pancreatic tissues were dehydrated and embedded in paraffin. All tissues were sectioned at 4 *μ*m. The routine process of staining was applied using hematoxylin and eosin stains [8]. The pancreatic sections were evaluated by light microscopy. Motic imaging software was used to evaluate the histological profile of pancreatic sections in all groups.

### 2.8. Statistical Analysis

All data were statistically subjected to Package for Social Sciences (SPSS for windows, version 22.0). The results were expressed as mean ± standard deviation (SD). Statistical analysis of one-way analysis of variance (ANOVA) followed by Dunnett's test were applied. Significance value was set at *P* less than 0.05.

## 3. Results

After extraction process of* O. oleaster* leaves,* J. procera* leaves, and* O*.* ficus-indica* stems, the yields of these plants were calculated. The yields means of* O*.* oleaster* leaves,* J. procera* leaves, and* O. ficus-indica* stems extracts were 21.3%, 17.7%, and 15.9%, respectively.

**Figure 1:**
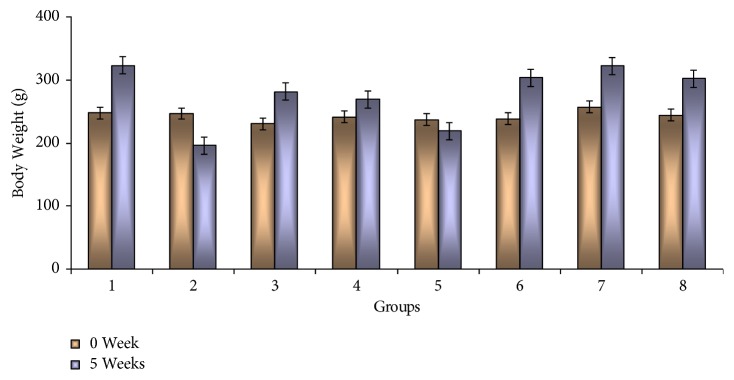
Changes of body weight after five weeks in control (group 1), STZ (group 2), STZ plus OLE (group 3), STZ plus JLE (group 4), STZ plus OSE (group 5), OLE (group 6), JLE (group 7), and OSE (group 8) treated rats.

Figure 1 shows mean body weight and mean body weight alterations (gain or loss) in all experimental groups after five weeks. A gradual increases in the body weight gain were detected in rats of groups 1, 6, 7, and 8, which amounted to + 30.5 %, + 27.3%, +25.4%, and + 23.5%, respectively. Significant decrease of body weight gain was observed in rats of group 2 (- 20.9 %) and group 5 (- 7.6%). The calculated percentage of body weight gain was + 22.1 in animals of group 3 and + 11.7% in rats of group 4.

The results of glucose, insulin, total protein, albumin, and globulin measurements are shown in Table 1. Glucose levels were significantly enhanced in rats of groups 2 (+ 410.2%,* P* < 0.000), 3 (+ 149.7%,* P* < 0.000), 4 (+ 264.9%,* P* < 0.000), and 5 (+ 160.2%,* P* < 0.001) compared to animals of group 1, while there were significant declines in glucose values of OLE (- 20.3%,* P* < 0.001), JLE (- 10.5%,* P* < 0.05), and OSE (- 19.8%,* P* < 0.05) treated normal rats. Statistical declines in the value of insulin were noted in diabetic animals of groups 2 (- 44.0%,* P* < 0.000), 3 (- 27.0%,* P* < 0.001), 4 (- 37.2%,* P* < 0.000), and 5 (- 33.2%,* P* < 0.001). The value of total protein was evoked in rats of group 2 (+ 19.5%,* P* < 0.01). Total proteins levels were statistically unchanged in rats of groups 3, 4, 5, 6, 7, and 8. Also, there was a notable decline in the values of albumin in animals of groups 2 (- 23.7,* P* < 0.05) and 4 (- 25.4,* P* < 0.05). On the other hand, insignificant changes of serum albumin were noted in rats of groups 3, 5, 6, 7, and 8. Notable elevations in the value of globulin were detected in rats of group 2 (+ 31.1%,* P* < 0.02) and group 5 (+ 14.0%,* P* < 0.05). Treatment of diabetic rats with OLE and JLE and normal rats with OLE, JLE and OSE (group 8) did not cause any significant alteration in the level of serum globulin.

Table 2 shows the lipids profile in all experimental groups. Relative to the normal control rats, the diabetic control rats of group 2 exhibited a significant enhancement in the level of triglycerides (+ 171.4%,* P* < 0.001). In addition, insignificant changes in the values of triglycerides were observed in diabetic (groups 3, 4 and 5) and nondiabetic (groups 6, 7, and 8) rats treated with OEL, JLE, and OSE compared with rats of group 1. The concentrations of cholesterol were remarkably increased in animals of group 2 (+ 48.4%,* P* < 0.01), 3 (+ 24.2%,* P* < 0.05), 4 (+ 17.9%,* P* < 0.02), and 5 (+ 29.5%,* P* < 0.02). OLE exposure to rats of group 6 significantly decreased the value of cholesterol (- 11.6%,* P* < 0.05), whereas the value of cholesterol was not changed in normal rats exposed to JEL (group 7) and OSE (group 8). The value of HDL-C was markedly decreased in rats of the second group (- 36.9%,* P* < 0.02), whereas the level of HDL-C was statistically increased in normal rats supplemented with OLE (+ 14.9%,* P* < 0.03). Insignificant alterations in the levels of HDL-C were noted in rats of groups 3, 4, 5, 7, and 8. The values of LDL-C (+ 44.1%,* P* < 0.01) and VLDL-C (+ 169.2%,* P* < 0.001) were notably increased in animals of group 2. Additionally, the values of LDL-C and VLDL-C were not significantly changed in diabetic and nondiabetic rats treated with OLE, JLE, and OSE

**Figure 2:**
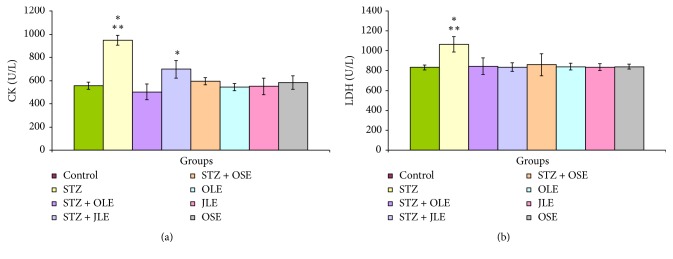
The levels of serum CK (a) and LDH (b) in control, STZ, STZ plus OLE, STZ plus JLE, STZ plus OSE, OLE, JLE, and OSE treated rats. ^*∗*^Significant difference between control and treated groups. ^*∗∗*^Significant difference between group 2 (STZ) and groups 3 (STZ + OLE), 4 (STZ + JLE), 5 (STZ + OSE), 6 (OLE), 7 (JLE), and 8 (OSE) treated rats.

The levels of CK and LDH are illustrated in Figures 2(a) and 2(b). Significant increases in the level of CK were noted in animals of group 2 (+ 70.5%,* P* < 0.001) and group 5 (+ 25.5%,* P* < 0.01). Insignificant changes in the values of CK were noted in rats of groups 3, 4, 6, 7, and 8 (Figure 2(a)). The value of LDH was significantly enhanced in animals of group 2 (+ 27.8%,* P* < 0.000). No statistically significant differences were noted in the values of LDH in rats of groups 3, 4, 5, 6, 7, and 8 compared to normal control rats (Figure 2(b)).

**Figure 3:**
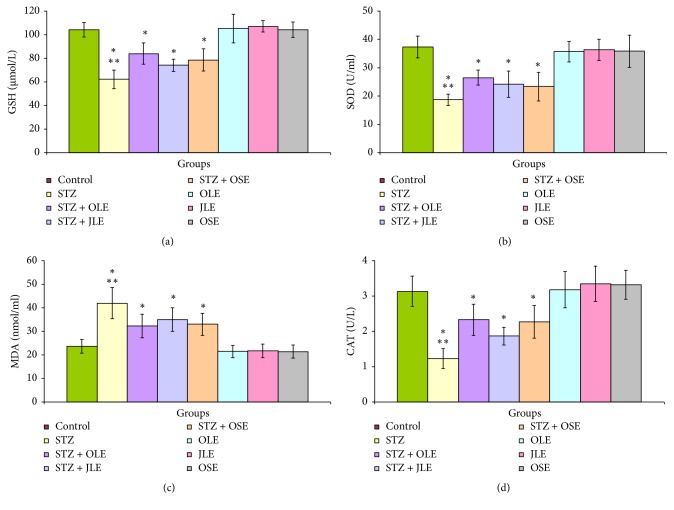
The levels of serum GSH (a), SOD (b), MDA (c), and CAT (d) in control, STZ, STZ plus OLE, STZ plus JLE, STZ plus OSE, OLE, JLE, and OSE treated rats. ^*∗*^Significant difference between control and treated groups. ^*∗∗*^Significant difference between group 2 (STZ) and groups 3 (STZ + OLE), 4 (STZ + JLE), 5 (STZ + OSE), 6 (OLE), 7 (JLE), and 8 (OSE) treated rats.

Figures 3(a)-3(d) represented the levels of GSH, SOD, MDA, and CAT, respectively, in all experimental groups. The levels of GSH were declined in diabetic rats of groups 2 (- 40.3%,* P* < 0.000), 3 (- 19.4%,* P* < 0.002), 4 (- 28.8%,* P* < 0.000), and 5 (- 24.6%,* P* < 0.005) (Figure 3(a)). Likewise, statistically there is a decrease in the levels serum SOD in diabetic rats of groups 2 (- 50.0%,* P* < 0.000), 3 (- 29.0%,* P* < 0.004), 4 (- 35.3%,* P* < 0.004), and 5 (- 37.5%,* P* < 0.006) (Figure 3(b)).  Figure 3(c) showed that the values of MDA were significantly increased in rats of groups 2 (+ 77.4%,* P* < 0.002), 3 (+ 36.6%,* P* < 0.03), 4 (+ 47.9%,* P* < 0.02), and 5 (+ 39.4%,* P* < 0.001). The values of CAT were declined in animals of group 2 (- 60.7%,* P* < 0.000), group 3 (- 25.6%,* P* < 0.02), group 4 (- 40.3%,* P* < 0.005), and group 5 (- 27.5%,* P* < 0.01). In addition, supplementation of OLE, JLE, and OSE to normal rats of groups 6, 7, and 8 showed insignificant alterations in the values of these oxidative stress markers.

**Figure 4:**
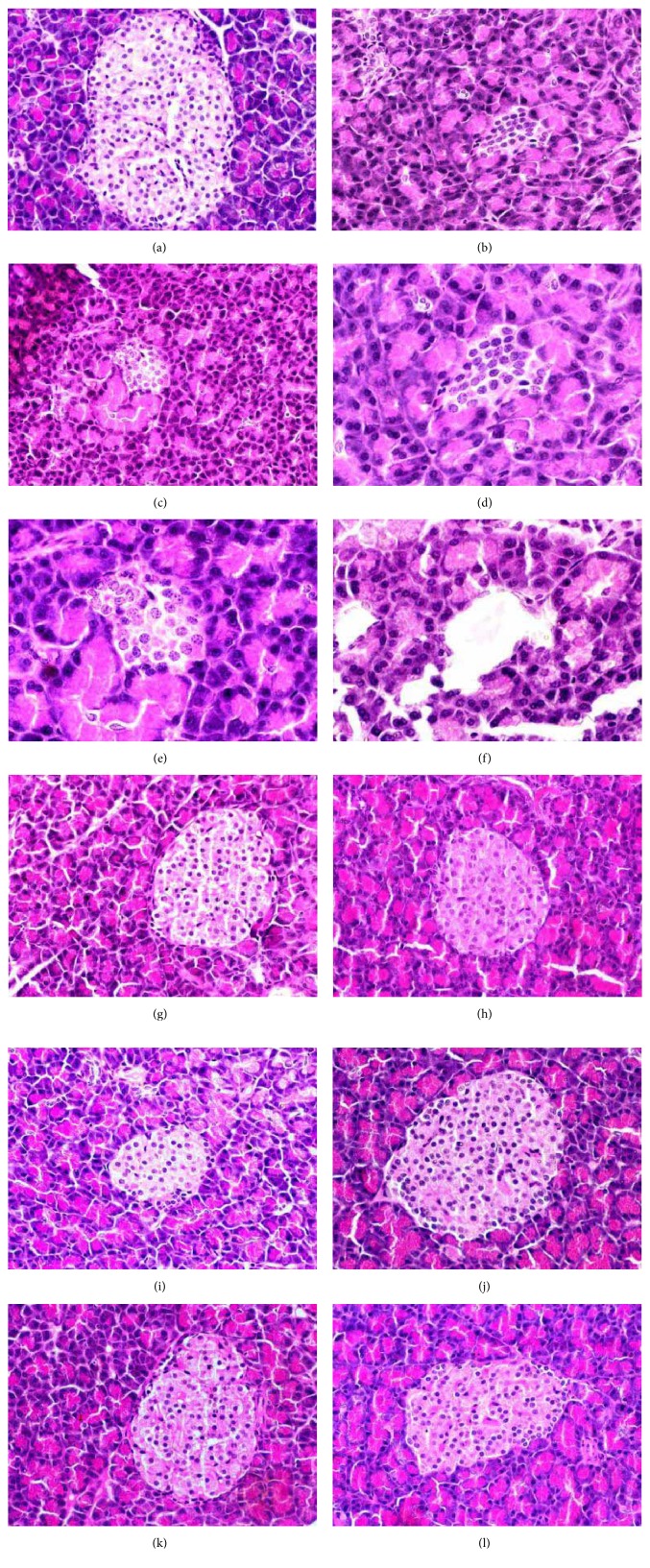
Photomicrographs of pancreas sections in each group. Normal pancreatic structure of control rats ((a) X200). STZ ((b) X200; (c-f) X400), STZ plus OLE ((g) X200), STZ plus JLE ((h) X200), STZ plus OSE ((i) X200), OLE ((j) X200), JLE ((k) X200), and OSE ((l) X200) treated rats.

Histopathological examination of pancreatic tissues form all experimental groups is illustrated in Figures 4(a)-4(l). As shown in Figures 4(a) (group 1), 4(j) (group 6), 4(k) (group 7), and 4(l) (group 8), normal pancreatic architectures including the normal cells of pancreatic (Langerhans) islet were seen. Pancreatic tissues of diabetic control rats (Figures 4(b)-4(f)) showed a decrease of Langerhans islet size and multiple degeneration and injuries. Furthermore, the number of *β*-cells was decreased, and some necrosis and destruction were noted. A mild size decreases and some degradation and injury of Langerhans islet were observed in STZ diabetic rats exposed to OLE (Figure 4(g)), JLE (Figure 4(h)), and OSE (Figure 4(i)).

## 4. Discussion

Many metabolic disturbances were associated with hyperglycemia in diabetic human [33]. The ability of insulin to mediate tissue glucose uptake is a major factor for glucose balance. Unfortunately, the use of synthetic insulin and oral glucose-lowering drugs have many side effects such as severe hypoglycemia at high doses, neurological disturbances, hepatic injury, headache, digestive disorder, lactic acidosis, and perhaps death. So, it is very important to look for new drugs with safe, cheap, and high efficiency properties for DM control instead of the current hypoglycemic drugs which associated with the side effects [34]. The present study was designed to examine the effect of OLE, JLE, and OSE on STZ-induced DM in Wistar male rats.

From the present results, it is obvious that the highly increased gain of body weight was noted in normal control rats followed by nondiabetic rats treated with OLE, JLE, and OSE and diabetic rats subjected to OLE and JLE. Highly significant decreases of body weight gain were noted in rats of groups 2 and 5. Rats of group 2 showed significant increases in values of glucose, insulin, total protein, globulin, triglycerides, cholesterol, LDL-C, and VLDL-C, whereas the levels of albumin and HDL-C were significantly declined. These findings are in agreement with other experimental diabetic investigations [8, 35–37].

The decline of body weight is attributed to the increase of blood glucose with inhibition of insulin level, decline of tissue proteins, and enhancement of muscle wasting in STZ diabetic animals [14, 38]. DM is accompanied with increased glycogenolysis, lipolysis, and gluconeogenesis and these biochemical activities result in muscles wasting and loss of tissue protein [39]. The body depends on insulin as a major anabolic hormone. The reduction and insufficiency of insulin caused metabolic disorders of glucose and also lipids and protein. The decrease and insufficiency of insulin converted anabolism to catabolism of proteins and lipids. Building of glucose depends on proteolysis and gluconeogenic amino acids by liver. Induction of negative nitrogen balance attributed to the catabolism of proteins and lipids; therefore the appetite and polyphagia were increased [40]. The present increase of serum glucose is confirmed by hypoinsulinemia and histopathological changes of pancreatic islets. Previous experimental investigations showed that the pancreatic tissues were damaged due to inductions of DM in animals. This damage included histopathological alterations of pancreatic islets accompanied with increase of blood glucose and decrease of insulin levels [8, 41–43].

The present alterations of serum proteins and lipids profiles indicate several disorders of the metabolism of protein and lipids in STZ-induced diabetes in rats. Hyperproteinemia and hypoalbuminemia and hyperglobulinemia attributed to hepatic and renal dysfunctions and losing of body water with high rates. Malawadi and Adiga [44] found that total protein and globulin levels were high and albumin levels were low in diabetic patients compared to controls. They reported that the elevation of total protein and globulin levels could be attributed to the elevation of various acute phase proteins, fibrinogen, and globulins in DM which contribute to the elevation in plasma proteins.

An elevation of blood triacylglycerol and cholesterol levels is a major indicator of body dyslipidemia which chronically leads to the increase of coronary heart injury [45]. Hyperlipidemia is one of the important factors associated with atherosclerosis, others being hypertension, smoking, DM, and other factors [46]. In DM, hyperlipidemia occurs due to increased lipolysis, leading to increased free fatty acids and glycerol which are taken up by liver to synthesize acetyl Co A. Acetyl Co A is a precursor for cholesterol synthesis. It has been reported that hyperlipidemia that occurs in STZ- induced diabetic rats is due to the increase in intestinal acyl coenzyme A activity [47].

The present study demonstrated that the values of CK and LDH were evoked in animals of group 2. The alteration of cardiac structure and function (cardiomyopathy) is one of DM complications. Diagnosis of cardiac enzymes is necessary for cardiomyopathy induced by DM. CK and LDH are commonly used as biomarkers for myocardial infarction. The values of these parameters were evoked as indicators of myocardial injury [48, 49]. However, it cannot be excluded that the present increase of serum CK and LDH levels may be attributed to their increase release form cardiac necrotic tissues in STZ diabetic rats. Necrosis and fibrosis of cardiac muscle fibers, systolic or diastolic disturbances, and alterations of cardiac biomarkers and oxidative stress markers were observed in diabetic patients and animals [50, 51].

The present significant decline of GSH, SOD, and CAT levels and an enhancement of MDA level confirmed that STZ induced oxidative stress. Previous studies showed that diabetic animals exhibited obviously changes in the values of these parameters [36, 52–54]. Significant increases in lipoperoxidation products and/or decreases of some antioxidants were observed in diabetic human and animals [55, 56]. Ansari et al. [57] suggested that the increase of MDA levels in diabetic rats was attributed to the increase levels of reactive oxygen species (ROS). Al-Attar and Alsalmi [8] showed that the values of GSH, SOD, and CAT were declined, and the value of MDA was significantly enhanced in diabetic animals.

From the present investigation, it is obvious that OLE, JLE, and OSE inhibited the physiological and histopathological alterations in STZ diabetic rats. Hierarchically, the present investigation showed that the most effective treatment was OLE followed by JLE and OSE. However, the possible mechanism of the studied extracts attributed to their antioxidant roles which evaluated by GSH, SOD, MDA, and CAT levels. Al-Attar and Alsalmi [8] studied the effect of leaves extract of olive (*Olea europaea*) on diabetic animals. They reported that the antidiabetic of this extract attributed to several factors such as the decrease of carbohydrates digestion rates and their absorption, the increase of hepatic glycogen formation, the inhibition of gluconeogenesis, the increase of insulin secretion and cellular uptake of glucose, insulin receptors improvement, and insulin resistance inhibition. Improvement of carbohydrate metabolism in diabetic rats supplemented with* J. phoenicea* extract was reported by Abdel-Rahim et al. [58]. El-Sawi et al. [59] investigated the influences of fruit and leaves of* J. phoenicea* on diabetic rats. The results showed that the extracts of* J. phoenicea* lowered the level blood glucose. Al-Ahdab [60] showed the extract of* J. phoenicea* declined blood glucose and MDA, enhanced the values of insulin, GSH and SOD, normalized serum levels of hepatic enzymes and biochemical parameters of renal function, and ameliorated lipid profile in STZ diabetic rats compared to untreated diabetic animals. Furthermore, an alleviation in the histopathological changes of pancreas was noted in diabetic rats exposed to* J. phoenicea* extract. Banerjee et al. [61] demonstrated that the methanolic extract of* J. communis* exhibited a significant and dose dependent reduction in the hyperglycaemic and hyperlipidemic conditions of STZ induced diabetic rats. Concerning* O*.* ficus-indica*, Yoon and Son [62] examined the effect of fruits and stems of* O*.* ficus-indica* on STZ-induced diabetic Sprague-Dawley male rats. They suggested that* O*.* ficus-indica* fruits and stems ameliorated blood glucose and metabolism of lipid in STZ-induced DM in rats. Hwang et al. [63] evaluated *α*-glucosidase inhibitory and antidiabetic effects of* O*.* ficus-indica* on streptozotocin STZ-induced diabetic Sprague-Dawley male rats. They showed that* O*.* ficus-indica* significantly improved deranged carbohydrate metabolism. However, the present study showed that OLE, JLE, and OSE attenuated the physiological and histopathological changes in STZ diabetic rats. Generally, the present obtained findings confirm that the influences of OLE, JLE, and OSE attributed to the antioxidant properties of their natural chemical constituents. Finally, this study indicates that OLE, JLE, and OSE may be a useful therapeutic factors for DM due to their antioxidant activities.

## 5. Conclusion

DM is one of the most important noninfective diseases to hit the globe in the present millennium. DM is one of the major complex and chronic disorders of carbohydrate, lipid, and protein metabolism. In spite of enormous advances in the field of medicine, there is no truly satisfactory drug for the treatment of DM. Presently, there is increasing evidence that many healthy natural food and medicinal plants and supplements have the potential to become valuable complementary therapy in the treatment of DM and its complications. The present study evaluated the hypoglycemic activities of OLE, JLE, and OSE extracts on diabetic male rats. Based on the present experimental data, it can be concluded that this study shows for the first time that OLE, JLE, and OSE extracts improve the physiological changes induced by STZ in the experimental animals. However, additional pharmacological, physiological, and biochemical studies are needed to clarify the optimum doses of these extracts as hypoglycemic factors and to elucidate their mechanisms of action.

## Figures and Tables

**Table 1:** The levels of serum glucose, insulin, total protein, albumin, and globulin of control, STZ, STZ plus OLE, STZ plus JLE, STZ plus OSE, OLE, JLE, and OSE treated rats after five weeks. Percentage changes are included in parentheses.

Treatments	Parameters				
	Glucose	Insulin	Total protein	Albumin	Globulin
	(mmol/L)	(*μ*IU/L)	(g/L)	(g/L)	(g/L)
Control	5.90±0.61	32.73±2.47	53.83±3.31	9.83±1.47	44.00±3.16
STZ	30.10±4.27^ab^	18.32±2.97^ab^	64.33±6.09^ab^	7.50±1.38^ab^	57.67±8.57^ab^
	(+ 410.2)	(- 44.0)	(+ 19.5)	(- 23.7)	(+ 31.1)
STZ + OLE	14.73±2.85^a^	23.88±2.96^a^	57.17±4.54	10.17±1.33	47.00±5.06
	(+ 149.7)	(- 27.0)	(+ 6.2)	(+ 3.5)	(+ 6.8)
STZ + JLE	21.53±3.97^a^	20.55±1.91^a^	54.33±6.38	7.33±1.51^a^	47.00±5.22
	(+ 264.9)	(- 37.2)	(+ 0.9)	(- 25.4)	(+ 6.8)
STZ + OSE	15.35±3.01^a^	21.87±1.94^a^	59.00±5.29	8.83±1.94	50.17±4.36^a^
	(+ 160.2)	(- 33.2)	(+ 9.6)	(- 10.2)	(+ 14.0)
OLE	4.70±0.37^a^	32.18±2.94	54.67±3.01	9.33±1.03	45.33±2.50
	(- 20.3)	(- 1.7)	(+ 1.6)	(- 5.1)	(+ 3.0)
JLE	5.28±0.34^a^	31.67±2.28	55.50±3.39	10.17±1.47	45.33±3.78
	(- 10.5)	(- 3.2)	(+ 3.1)	(+ 1.33)	(+ 3.0)
OSE	4.73±0.26^a^	32.87±3.18	54.60±2.80	9.67±1.63	45.00±3.10
	(- 19.8)	(+ 0.4)	(+ 1.4)	(- 1.6)	(+ 2.3)

Data represent the means ± SD of 6 animals per group.  ^a^Significant difference between control and treated groups.  ^b^Significant difference between group 2 (STZ) and groups 3 (STZ + OLE), 4 (STZ + JLE), 5 (STZ + OSE), 6 (OLE), 7 (JLE), and 8 (OSE) treated rats.

**Table 2:** The levels of serum triglycerides, cholesterol, HDL-C, LDL-C, and VLDL-C of control, STZ, STZ plus OLE, STZ plus JLE, STZ plus OSE, OLE, JLE, and OSE treated rats after five weeks. Percentage changes are included in parentheses.

Treatments	Parameters				
	Triglycerides	Cholesterol	HDL-C	LDL-C	VLDL-C
	(mmol/L)	(mmol/L)	(mmol/L)	(mmol/L)	(mmol/L)
Control	0.56±0.06	0.95±0.06	1.41±0.15	0.34±0.05	0.26±0.03
STZ	1.52±0.33^ab^	1.41±0.24^ab^	0.89±0.19^ab^	0.49±0.09^ab^	0.70±0.15^ab^
	(+ 171.4)	(+ 48.4)	(- 36.9)	(+ 44.1)	(+ 169.2)
STZ + OLE	0.55±0.17	1.18±0.18^a^	1.34±0.18	0.37±0.10	0.25±0.08
	(- 1.8)	(+ 24.2)	(- 5.0)	(+ 8.8)	(- 3.9)
STZ + JLE	0.68±0.36	1.12±0.15^a^	1.29±0.35	0.29±0.05	0.31±0.17
	(+ 21.4)	(+ 17.9)	(- 8.5)	(- 14.7)	(+ 19.2)
STZ + OSE	0.92±0.34	1.23±0.23^a^	1.54±0.32	0.35±0.12	0.42±0.16
	(+ 73.2)	(+ 29.5)	(+ 9.2)	(+ 2.9)	(- 73.1)
OLE	0.48±0.09	0.84±0.12^a^	1.62±0.15^a^	0.36±0.09	0.22±0.04
	(- 14.3)	(- 11.6)	(+ 14.9)	(+ 5.9)	(- 15.4)
JLE	0.58±0.10	0.92±0.11	1.47±0.21	0.36±0.05	0.27±0.05
	(+ 3.6)	(- 3.2)	(+ 4.3)	(+ 5.9)	(+ 3.9)
OSE	0.52±0.07	0.90±0.17	1.46±0.25	0.29±0.04	0.24±0.03
	(- 7.1)	(- 5.3)	(+ 3.6)	(- 14.7)	(- 11.5)

Data represent the means ± SD of 6 animals per group.  ^a^Significant difference between control and treated groups.  ^b^Significant difference between group 2 (STZ) and groups 3 (STZ + OLE), 4 (STZ + JLE), 5 (STZ + OSE), 6 (OLE), 7 (JLE), and 8 (OSE) treated rats.

## Data Availability

All relevant data are within the manuscript. All data were statistically analyzed as mentioned in the submitted manuscript.

## Abbreviations

CAT: Catalase
CK: Creatine kinase
DM: Diabetes mellitus
GSH: Glutathione
HDL-C: High density lipoprotein cholesterol
JLE: * Juniperus procera* leaves extract
LDH: Lactate dehydrogenase
LDL-C: Low density lipoprotein cholesterol
MDA: Malondialdehyde
OLE: * Olea oleaster* leaves extract
OSE: * Opuntia ficus-indica* stems extract
ROS: Reactive oxygen species
SOD: Superoxide dismutase
STZ: Streptozotocin
VLDL-C: Very low density lipoprotein cholesterol.
